# Current Applications and Future Prospects of Fecal Microbiota Transplantation

**DOI:** 10.14789/ejmj.JMJ24-0045-R

**Published:** 2025-03-12

**Authors:** DAI ISHIKAWA, XIAOCHEN ZHANG, AKIHITO NAGAHARA

**Affiliations:** 1 Department of Gastroenterology, Juntendo University School of Medicine, Tokyo, Japan; 1 Department of Gastroenterology, Juntendo University School of Medicine, Tokyo, Japan; 2 Innovative Microbiome Therapy Research Center, Juntendo University Graduate School of Medicine, Tokyo, Japan; 2 Innovative Microbiome Therapy Research Center, Juntendo University Graduate School of Medicine, Tokyo, Japan

**Keywords:** fecal microbiota transplantation, *Clostridioides difficile* infection, ulcerative colitis, applications, dysbiosis

## Abstract

In recent years, the impact of gut microbiota on human health has gained increasing recognition, highlighting the potential benefits of regulating gut microbiota for patient care. Consequently, fecal microbiota transplantation (FMT) has emerged as a novel treatment for improving dysbiosis. It is necessary to summarize and discuss the current research and future development possibilities.

This advanced microbial therapy restores the gut microbiome by introducing a diverse array of microorganisms from healthy donors, thereby correcting dysbiosis and re-establishing a fully functional ecosystem. Ongoing research on FMT is actively conducted in various fields worldwide. This study provides an overview of the progress of clinical research on the effects of FMT in gastrointestinal diseases, immune checkpoint inhibitors, allergic diseases, and central nervous system diseases, as well as the results of our ongoing clinical study on “FMT combined with antibiotics for ulcerative colitis.”

## Introduction

Advancements in gut microbiota research have revealed that dysbiosis ─ an imbalance in the gut microbiome ─ is involved not only in the development of gastrointestinal diseases such as inflammatory bowel disease (IBD) and irritable bowel syndrome (IBS), but also in other conditions, including metabolic diseases such as obesity and diabetes, autoimmune diseases such as rheumatoid arthritis and multiple sclerosis, and mental disorders such as autism and depression^1)^. Fecal microbiota transplantation (FMT) is a novel therapy that restores gut health by introducing a diverse range of healthy microbes to correct the imbalance^2)^.

In 2013, FMT was reported to be highly effective in treating *Clostridioides difficile infections* (CDIs)^3)^, which are typically caused by long-term antibiotic use. FMT is now strongly recommended in the treatment guidelines issued by the United States and Europe for recurrent CDI^4, 5)^, with a reported success rate of 92%^6)^. In Japan, the increasing prevalence of ulcerative colitis (UC) has heightened the expectations for FMT as a new treatment option. Although current research findings remain inconsistent, the First International Rome Consensus Conference on FMT in IBD aimed to establish FMT as a validated treatment approach and highlight its potential as an effective therapy for IBD^7)^. Furthermore, FMT was approved as an Advanced Medical Care B program for UC treatment in January 2023 (https://www.metagentx.com/en/news/230104_senshinb/). This study aimed to provide an overview of the current status of FMT and present the results of our ongoing clinical study on “FMT combined with antibiotics.”

## Established application of FMT in CDI

Improper antibiotic use can cause dysbiosis, which increases the risk of CDI^8)^. CDI has also gradually shifted from being predominantly a nosocomial condition, with >41% of cases currently being community acquired^9)^. In Japan, vancomycin (VCM) and metronidazole (MNZ) are the primary treatments; however, refractory cases are encountered in clinical practice^10)^. Patients with prolonged antibiotic use, long-term hospitalization, advanced age (>65 years), or other diseases are particularly prone to recurrent or refractory CDI^8)^. The earliest literature on FMT for refractory CDI dates back to 1958. Eiseman et al.^11)^ reported four patients with recurrent pseudomembranous colitis. FMT was performed by administering one to three enemas. None of the patients showed improvements in symptoms or reported side effects. Although some individual case reports have been published since then, no systematic studies have been conducted. In 2013, van Nood et al.^3)^ conducted a randomized controlled trial (RCT) comparing FMT with standard treatment (VCM). The trial reported an 81% cure rate after a single FMT (13 out of 16 patients) and a 67% cure rate (2 out of 3) in previously unresponsive patients after another FMT from a different donor, culminating in an impressive total cure rate of 94%. Furthermore, the analysis of intestinal microbiota revealed improved diversity after FMT, closely resembling that of healthy donors. Based on the these findings, the European and American CDI treatment guidelines^4, 12)^ recommended for patients with recurrent CDI who are unresponsive to VCM. In Europe and the United States, the emergence of nosocomial infection caused by an antimicrobial-resistant North American Pulsed-field gel electrophoresis type 1 (NAP1) strain has exacerbated the issue of nosocomial infections, with NAP1 detected in 30% of patients with hospital- acquired infections^13)^. The rising prevalence of drug-resistant and refractory CDI and the worsening of the disease have made the FMT an increasingly important drug-independent intestinal microbiome therapy. Although Japan has not encountered severe CDI issues, globalization necessitates the strengthening of the country’s FMT implementation systems to address potential future challenges effectively.

## Exploratory applications of FMT in IBD

In Japan, the number of patients with UC has been steadily increasing^14)^. Although the emergence of biologics and immunosuppressants has improved remission rates, patients are compelled to use these medications in the long-term owing to the absence of standardized discontinuation criteria. The use of these drugs is challenging for pediatric and older patients due to immune tolerance issues and the significant risk of developing side effects^15, 16)^. Therefore, a fundamental treatment method with fewer side effects is needed. As UC is a representative disease associated with dysbiosis, FMT has emerged as a promising treatment option for UC^2)^.

The earliest study investigating the use of FMT for UC was conducted by Bennet et al. in 1989^17)^, who administered a single FMT enema following antibiotic treatment, which resulted in patient remission. Subsequent studies have the reported the effectiveness of FMT in UC treatment; however, most of these studies are case reports, and the therapeutic effects remain a topic for discussion.

To date, six double-blind RCTs on FMT for UC have been reported (Table 1). In May 2015, two RCTs^18, 19)^ on FMT for UC were published by the American journal *Gastroenterology*. In a study by Moayyedi et al., the FMT group with UC received 50 mL of healthy donor stool via enema once a week for six consecutive weeks, while the placebo group received water. The results revealed that the remission rate in the FMT group (24%) was significantly higher than that in the placebo group (5%). In the study by Rossen et al., patients with UC were allocated into two groups: the FMT group, which received donor stool via a nasoduodenal tube, and the control group, which received autologous stool. Both groups underwent a total of two treatments at weeks 0 and 3. The results indicated that the remission rate in the FMT group (30.4%) was not significantly different from that in the control group (20.0%), thus failing to validate the efficacy of FMT in UC. These two RCTs were the first to investigate FMT for UC, but the conflicting results raised concerns. This discrepancy may be attributed to the significant differences in the administration routes and frequencies of FMT. Although FMT has demonstrated high success rates in treating CDI, regardless of the administration method, these results indicate the need for a standardized FMT protocol for UC. Analysis of the gut microbiota revealed the recovery of diversity and similarity between the donor and recipient microbiota. Notably, Rossen et al.^18)^ reported that remission was associated with butyrate-producing bacteria, specifically *Clostridium* clusters IV and XIVa. However, similar changes in the gut microbiota were also observed among responsive patients in the placebo group. This result suggests that these microbiota changes are part of the improvement, rather than the mechanisms of the therapeutic effect. Therefore, the precise mechanism of action FMT remains unclear.

Paramsothy et al.^20)^ reported the third RCT on FMT for UC in February 2017. This study increased the diversity of donor stools by mixing stool samples from three to seven donors. The patients underwent FMT via colonoscopy, followed by self-administered enemas five times per week for 8 weeks, totaling 41 FMT treatments. Despite the use of stringent evaluation criteria (requiring both clinical and endoscopic remissions while gradually tapering corticosteroids), the FMT group achieved a 27% remission rate, which was significantly higher than the 8% remission rate in the isotonic saline control group. However, the complexity of this treatment method, which involves 40 self-administered enemas using frozen donor stool, raises concerns about its practicality as a treatment approach. In 2019, Costello et al.^21)^ reported successful remission after only three FMT administrations using a solution prepared under anaerobic conditions. The latest RCT, conducted in 2021^22)^, used freeze-dried donor stool capsules for oral intake. After taking the three types of antibiotics for two weeks, the patients continued to take the capsules for eight weeks, achieving a 73% clinical remission rate, which was a remarkable result. In the same year, Crother et al.^23)^ published a study involving 12 patients with UC. Despite performing 85 FMT procedures, only two of the six patients in the FMT group achieved remission by week 12, revealing no significant difference compared with the placebo group. Although the conclusions of the six RCTs were inconsistent, a network meta-analysis comparing FMT with five biologics currently used as standard therapy for severe refractory UC revealed that FMT is comparable to biologics in terms of remission and symptom improvement rates^24)^.

Only one RCT^25)^ reported the use of FMT in the treatment of Crohn’s Disease (CD), but the findings were not promising, as no significant difference was observed in the remission rates between the FMT and control groups. This outcome may be related to the characteristics of CD lesions, which can extend into the small intestine, rendering their administration more difficult. Therefore, the location of the lesion, whether in the colon or the small intestine, may be a critical factor influencing remission, highlighting the need for further research to control variables such as lesion location.

**Table 1:** Double-blind RCT of FMT for UC

Author, year	Rossen et al. 2015^18)^	Moayyedi et al. 2015^19)^	Paramsothy et al. 2017^20)^	Costello et al. 2019^21)^	Haifer et al. 2021^22)^	Crothers et al. 2021^23)^
Patients No.	48 (FMT:23, placebo:25)	75 (FMT:38, placebo:37)	81 (FMT:41, placebo:40)	73 (FMT:38, placebo:35)	35(FMT:15, placebo:20)	12 (FMT:6, placebo:6)
Pretreatment	Bowel lavage	none	Bowel lavage	Bowel lavage	Amoxicillin, doxycycline, metronidazole.	Ciprofloxacin, metronidazole & Bowel lavage
FMT times	2 times	6 times	41 times	3 times	49 times	85 times
Route of administration	500ml Duodenal infusions	50ml enema	150ml colonoscopy, enema	200ml colonoscopy, 100ml enema	6 lyophilized oral capsules	120ml colonoscopy, 550μl oral capsule
Regimen of administration	2 nasoduodenal infusions 3 weeks apart	Weekly enemas for 6 weeks	Colonoscopic infusion followed by enemas 5 times/ week for 8 weeks	Colonoscopic infusion followed by 2 enemas in 1week	First week: 4 times a day. second week: Twice daily. Followed 6 weeks: once daily.	Colonoscopic infusion followed by oral capsule daily for 12weeks
Placebo	autologous	water	processed isotonic saline	autologous	double encapsulated	sham
Donor	single	single	multiple (3-7 donor)	multiple (3-4 donor)	single	single
Stool	fresh	fresh/ frozen	frozen −80°C	frozen −80°C	lyophilised	frozen −20°C
Primary endpoint (FMT vs. placebo)	CR+ER at week 12 30% vs. 20%, p=0.51	CR+ER at week 7 24% vs. 5%, p=0.03	CR+ER/Er at week 8 27% vs. 8%, p=0.02	CR+ER at week 8 32% vs. 9%, p=0.03	CR+ER/Er at week 8 53% vs. 15%, p=0.027	CR at week 12 2/6 vs. 0/6, p=0.45

FMT: fecal microbiota transplantation, CR: Clinical remission, ER: Colonoscopic remission, Er: Colonoscopic response, RCT: randomized controlled trial, UC: ulcerative colitis.

## Our team’s application of FMT in UC

The human gut microbiota^26)^ consists of up to 3.8×10^13^ trillion bacteria, and FMT in isolation cannot ensure efficient colonization of donor bacteria, limiting its ability to effectively improve gut dysbiosis. To overcome this challenge, we administered three oral antibiotics ─ amoxicillin, fosfomycin, and MNZ (AFM) ─ for two weeks prior to FMT. This approach aimed to reduce the recipient’s original gut microbiota, thereby facilitating the efficient colonization of the transplanted bacteria. This combination therapy is referred to as “antibiotic- FMT” (A-FMT).

The use of antibiotic-assisted FMT therapy has been documented in a previous study. Borody et al.^27)^ reported a high cure rate using VCM, rifampicin, and MNZ as pre-treatments, followed by five sessions of FMT. To establish a more effective FMT protocol for the treatment of UC, we modified an antibiotic therapy (amoxicillin, tetracycline, and MNZ; ATM) that was previously reported to be effective for UC^28)^ by replacing tetracycline with fosfomycin and proposed the A-FMT protocol. We treated 41 patients with UC^29)^, of whom 21 received A-FMT and 20 received AFM alone. The results revealed that the proportion of Bacteroidetes significantly decreased after AFM therapy. By the 4th week after FMT, the Bacteroidetes levels had significantly recovered in patients who responded to treatment, while no recovery was observed in non-responders. By contrast, the AFM-alone group demonstrated insufficient recovery after 4 weeks, with no observed correlation to the treatment outcomes. These findings suggest a strong association between Bacteroidetes recovery and the efficacy of A-FMT. A subsequent species-level analysis of Bacteroidetes demonstrated that responders experienced improved species-level diversity after A-FMT, with their gut microbiota composition becoming more similar to that of the donor^30)^. This outcome marked the first instance that antibiotic pre-treatment in AFM therapy facilitated the efficient colonization of donor gut microbiota, with successful colonization observed among responders.

### Donor matching and long-term responders

Increasing evidence highlighted the critical role of compatibility between donor and recipient microbiomes in achieving a sustained response through cross-individual microbiota interactions^31, 32)^. Donor- patient matching has emerged as a pivotal factor influencing the efficacy of A-FMT, paving the way for the development of stratified FMT approaches and microbiome-based drug discovery. In another study^33)^, short-term therapeutic outcomes did not reveal a correlation between the donor-patient relationship and therapeutic success. However, a 2-year long-term follow-up revealed that sibling transplantation and a donor-patient age difference of less than 10 years were associated with long-term remission. Notably, in a case where the younger brother served as the donor, a single A-FMT treatment resulted in a 2-year sustained remission. A species-level gut microbiota analysis revealed efficient and stable transplantation of the donor’s gut microbiota, particularly the Bacteroidota species, into the sibling’s gut, with minimal changes over the 2 years. Knoll et al.^34)^ have also reported that microbiota reshaping therapy using siblings as donors is a viable treatment option for children with UC. The sibling’s gut microbiota closely resemble that of the patient’s microbiota prior to disease onset, whereas dysbiosis creates a structural difference between the patient’s microbiota and that of their siblings. Transplanting a microbiota with a composition similar to that in the pre-disease state may facilitate the patients to return to their pre-disease condition and achieve more stable colonization. Therefore, we propose that these siblings could serve as optimal donors for microbiota reshaping therapy for IBD.

## Expansion of FMT indications

The indications for FMT are not limited to the treatment of rCDI and UC. The processing of fecal solutions used in FMT promotes the development of microbiome-based drugs (fecal-derived preparations), which can broaden its applications to treat a wider range of diseases.

### Application of FMT and immune checkpoint inhibitors

In 2020, *Science* published a case study involving 10 patients with anti-programmed cell death 1 (PD-1) resistant metastatic melanoma, who were treated with FMT followed by re-administration of anti-PD-1 therapy. The results revealed partial efficacy in two patients and significant efficacy in one^35)^. In the same year, Science published another clinical study^36)^ evaluating the safety and efficacy of FMT combined with anti-PD-1 in patients with PD-1-refractory melanoma. A recent review^37)^ indicated that 37.5% of patients with melanoma who were previously resistant to immune checkpoint inhibitors (ICI) immunotherapy exhibited complete or partial response after FMT and subsequent immunotherapy. No serious FMT-related adverse events were reported.

These findings underscore the potential of FMT and suggest a significant association between gut bacteria and cancer immunity, paving the way for the development of microbiome-based therapeutics.

### Application of FMT and allergic diseases

Exposure to the infant microbiome shortly after birth is crucial for the development of protective immunity and immune tolerance. Disruption of microbial exposure may increase vulnerability to infections in newborns or contribute to a higher risk of developing allergies and autoimmune diseases^38)^. Similarly, changes in the gut microbiota of infants after birth have been associated with the development of allergies^39)^. However, no consensus exists regarding which microbes play a protective role or are associated with an increased risk of developing allergies.

Infants born via vaginal delivery are colonized by their mother’s gut microbiota^40, 41)^, whereas those born via cesarean section are less exposed to these bacteria^41)^, leading to differences in the composition of their microbiomes^42)^. A study that performed FMT using diluted maternal feces from newborns delivered via cesarean section found significant similarities to the gut microbiota of infants born through vaginal delivery^43)^. This finding suggests that, unlike in adult patients, FMT can induce significant changes in the gut microbiota of infants, making it a potential strategy for preventing allergies. Therefore, A-FMT may be an effective treatment option for pediatric patients with allergic diseases.

### Application of FMT and central nervous system diseases

In recent years, the increasing prevalence of autism spectrum disorder (ASD) worldwide has garnered significant attention^44)^. The etiology of ASD remains unclear, and no effective treatment is available^45)^. ASD, a neurodevelopmental disorder in children, is associated with gut microbiota dysbiosis^46)^. In addition to abnormal neurological behaviors, children with ASD typically exhibit significant gastrointestinal symptoms^47)^. The gut microbiota influences brain development and behavior through the gut-brain axis^48)^. FMT has been reported to improve both behavioral and gastrointestinal symptoms in ASD by modulating the gut microbiota^49)^.

Furthermore, several studies^50-52)^ have linked the gut microbiota to various neurological disorders. Animal experiments in mice have shown a potential link between gut microbiota composition changes and Alzheimer’s disease (AD)^53)^. An 82-year-old male patient with AD demonstrated improvements in cognitive function, memory, and mood after receiving FMT from his 85-year-old wife^54)^. Additionally, a 90-year-old female with AD and severe CDI underwent FMT from a 27-year-old healthy male donor, resulting in improved cognitive function, microbiota diversity, and Short-Chain Fatty Acids production^55)^. Nevertheless, related studies remain limited. The therapeutic potential of FMT in multiple neurological disorders warrants further investigation.

### Application of FMT and metabolic disease

The gut microbiota is one of the key regulatory systems for energy and substrate metabolism in the human body. Dysbiosis of the gut microbiota can lead to metabolic imbalances, contributing to chronic inflammation, insulin resistance, obesity, and other metabolic disorders. Type 2 diabetes mellitus (T2DM) is a chronic metabolic disease characterized by hyperglycemia, affecting 537 million adults globally^56)^. Modulating the composition of the gut microbiota in patients with T2DM may offer a novel therapeutic approach. In T2DM mice receiving FMT, HbA1c levels decreased, inflammatory factors such as Interleukin-6 (IL-6) and Tumor Necrosis Factor Alpha (TNF-α) were reduced, and both pancreatic islet function and insulin sensitivity improved^57)^. In clinical trials, FMT-treated obese patients with T2DM acquired ≥20% lean-associated microbiota by week24^58)^. Another study demonstrated that the gut microbiota of patients with T2DM was remodeled after FMT, leading to significant reductions in blood glucose and glycated hemoglobin levels^59)^. By increasing beneficial bacterial species and altering the gut microbiota composition, FMT may play a regulatory role in metabolism for patients with T2DM.

Non-alcoholic fatty liver disease (NAFLD) is a metabolic disorder characterized by the accumulation of fat in the liver^60)^. The number of preclinical and clinical studies evaluating the efficacy of FMT in the treatment of NAFLD has been increasing. Additionally, the role of gut microbiota in patients with other liver diseases has also attracted attention.^61)^. Nevertheless, the limited number of studies investigating the use of FMT in the treatment of liver diseases is insufficient to draw definitive conclusions.

### Future outlook of FMT

Around the world, research is intensively focused on the impact of FMT on various disease areas. Therefore, it is highly likely that in the future, the best stool donors will be selected based on individual patient conditions and disease information, which is also what we hope to see. However, this tailored FMT requires determining the optimal conditions for matching donors with recipients, identifying and selecting specific microbes or microbial combinations with therapeutic potential, and establishing global donor screening standards to ensure safety and stability. Additionally, long-term efficacy and safety of personalized FMT treatment need to be tracked.

If the key baceria or super-donors essential for treatment can be identified, it may become possible to simulate the microbial environment of super-donor feces and produce artificial feces. This could then be administered to patients, along with key microbial strains ─ similar to the widely recognized lactic acid bacteria ─ through enhanced oral supplementation. This approach would improve both the psychological and physical acceptance of FMT by patients while reducing the risk of exposure to infectious diseases. This is a path that requires rigorous and rapid development.

## Conclusion

Research on FMT is actively being conducted worldwide. In the United States, FMT has progressed to the clinical application stage. In November 2022, the Ferring Pharmaceuticals’ microbiota-based enema solution, REBYOTA, received approval from regulatory authorities for the treatment of CDI. In Australia, BiomeBank’s FMT solution for CDI has also received regulatory approval. Our research team is committed to establishing an effective and safe foundation for FMT in Japan. As of March 2023, our institution has enrolled approximately 240 patients with UC and 240 donors participating in our study. We aim to clarify the therapeutic mechanisms and explore more effective treatment methods through multi-faceted analysis of the accumulated data. This research will also significantly contribute to the establishment of “fundamental gut microbiota therapy” for refractory diseases and other conditions associated with dysbiosis.

## Funding

The authors received no financial support for the research.

## Author contributions

DI provided the initial draft concept, and XZ completed the writing of the detailed content. The manuscript was subsequently reviewed and approved by DI and AN All authors read and approved the final manuscript.

## Conflicts of interest statement

The authors declare that there are no conflicts of interest.

## Availability of data and materials

Not applicable.

## References

[1] Choi HH, Cho YS: Fecal microbiota transplantation: Current applications, effectiveness, and future perspectives. Clin Endosc, 2016; 49: 257-265. 26956193 10.5946/ce.2015.117 PMC4895930

[2] Borody TJ, Paramsothy S, Agrawal G: Fecal microbiota transplantation: Indications, methods, evidence, and future directions. Curr Gastroenterol Rep, 2013; 15: 337. 23852569 10.1007/s11894-013-0337-1 PMC3742951

[3] van Nood E, Vrieze A, Nieuwdorp M, et al: Duodenal infusion of donor feces for recurrent Clostridium difficile. N Engl J Med, 2013; 368: 407-415. 23323867 10.1056/NEJMoa1205037

[4] Cammarota G, Ianiro G, Tilg H, et al: European consensus conference on faecal microbiota transplantation in clinical practice. Gut, 2017; 66: 569-580. 28087657 10.1136/gutjnl-2016-313017 PMC5529972

[5] McDonald LC, Gerding DN, Johnson S, et al: Clinical practice guidelines for Clostridium difficile infection in adults and children: 2017 update by the Infectious Diseases Society of America (IDSA) and Society for Healthcare Epidemiology of America (SHEA). Clin Infect Dis, 2018; 66: e1-e48. 29462280 10.1093/cid/cix1085 PMC6018983

[6] Quraishi MN, Widlak M, Bhala N, et al: Systematic review with meta-analysis: The efficacy of faecal microbiota transplantation for the treatment of recurrent and refractory Clostridium difficile infection. Aliment Pharmacol Ther, 2017; 46: 479-493. 28707337 10.1111/apt.14201

[7] Lopetuso LR, Deleu S, Godny L, et al: The first international Rome consensus conference on gut microbiota and faecal microbiota transplantation in inflammatory bowel disease. Gut, 2023; 72: 1642-1650. 37339849 10.1136/gutjnl-2023-329948 PMC10423477

[8] Cymbal M, Chatterjee A, Baggott B, Auron M: Management of Clostridioides difficile infection: Diagnosis, treatment, and future perspectives. Am J Med, 2024; 137: 571-576. 38508330 10.1016/j.amjmed.2024.03.024

[9] Guery B, Galperine T, Barbut F: Clostridioides difficile: diagnosis and treatments. BMJ, 2019; 366: l4609. 31431428 10.1136/bmj.l4609

[10] Riley TV, Kimura T: The epidemiology of Clostridium difficile infection in Japan: a systematic review. Infect Dis Ther, 2018; 7: 39-70. 29441500 10.1007/s40121-018-0186-1 PMC5840105

[11] Eiseman B, Silen W, Bascom GS, Kauvar AJ: Fecal enema as an adjunct in the treatment of pseudomembranous enterocolitis. Surgery, 1958; 44: 854-859. 13592638

[12] McDonald LC, Gerding DN, Johnson S, et al: Clinical practice guidelines for Clostridium difficile infection in adults and children: 2017 update by the Infectious Diseases Society of America (IDSA) and Society for Healthcare Epidemiology of America (SHEA). Clin Infect Dis, 2018; 66: 987-994. 29562266 10.1093/cid/ciy149

[13] Czepiel J, Dróżdż M, Pituch H, et al: Clostridium difficile infection: Review. Eur J Clin Microbiol Infect Dis, 2019; 38: 1211-1221. 30945014 10.1007/s10096-019-03539-6 PMC6570665

[14] Okabayashi S, Kobayashi T, Hibi T: Inflammatory bowel disease in Japan — Is it similar to or different from Westerns? J Anus Rectum Colon, 2020; 4: 1-13. 32002471 10.23922/jarc.2019-003 PMC6989123

[15] Stallmach A, Hagel S, Bruns T: Adverse effects of biologics used for treating IBD. Best Pract Res Clin Gastroenterol, 2010; 24: 167-182. 20227030 10.1016/j.bpg.2010.01.002

[16] Zenlea T, Peppercorn MA: Immunosuppressive therapies for inflammatory bowel disease. World J Gastroenterol, 2014; 20: 3146-3152. 10.3748/wjg.v20.i12.3146 PMC3964386 24696600

[17] Bennet JD, Brinkman M: Treatment of ulcerative colitis by implantation of normal colonic flora. Lancet, 1989; 1: 164. 10.1016/s0140-6736(89)91183-5 2563083

[18] Rossen NG, Fuentes S, van der Spek MJ, et al: Findings from a randomized controlled trial of fecal transplantation for patients with ulcerative colitis. Gastroenterology, 2015; 149: 110-118. e4. 25836986 10.1053/j.gastro.2015.03.045

[19] Moayyedi P, Surette MG, Kim PT, et al: Fecal microbiota transplantation induces remission in patients with active ulcerative colitis in a randomized controlled trial. Gastroenterology, 2015; 149: 102-109. e6. 25857665 10.1053/j.gastro.2015.04.001

[20] Paramsothy S, Kamm MA, Kaakoush NO, et al: Multidonor intensive faecal microbiota transplantation for active ulcerative colitis: a randomised placebo-controlled trial. Lancet, 2017; 389: 1218-1228. 28214091 10.1016/S0140-6736(17)30182-4

[21] Costello SP, Hughes PA, Waters O, et al: Effect of fecal microbiota transplantation on 8-week remission in patients with ulcerative colitis: a randomized clinical trial. JAMA, 2019; 321: 156-164. 30644982 10.1001/jama.2018.20046 PMC6439766

[22] Haifer C, Paramsothy S, Kaakoush NO, et al: Lyophilised oral faecal microbiota transplantation for ulcerative colitis (LOTUS): a randomised, double-blind, placebo-controlled trial. Lancet Gastroenterol Hepatol, 2022; 7: 141-151. 34863330 10.1016/S2468-1253(21)00400-3

[23] Crothers JW, Chu ND, Nguyen LTT, et al: Daily, oral FMT for long-term maintenance therapy in ulcerative colitis: results of a single-center, prospective, randomized pilot study. BMC Gastroenterol, 2021; 21: 281. 10.1186/s12876-021-01856-9 PMC8268596 34238227

[24] Zhou HY, Guo B, Lufumpa E, et al: Comparative of the effectiveness and safety of biological agents, tofacitinib, and fecal microbiota transplantation in ulcerative colitis: systematic review and network meta-analysis. Immunol Invest, 2021; 50: 323-337. 32009472 10.1080/08820139.2020.1714650

[25] Sokol H, Landman C, Seksik P, et al: Fecal microbiota transplantation to maintain remission in Crohn’s disease: a pilot randomized controlled study. Microbiome, 2020; 8: 12. 32014035 10.1186/s40168-020-0792-5 PMC6998149

[26] Sender R, Fuchs S, Milo R: Revised estimates for the number of human and bacteria cells in the body. PLoS Biol, 2016; 14: e1002533. 27541692 10.1371/journal.pbio.1002533 PMC4991899

[27] Borody TJ, Warren EF, Leis S, Surace R, Ashman O: Treatment of ulcerative colitis using fecal bacteriotherapy. J Clin Gastroenterol, 2003; 37: 42-47. 12811208 10.1097/00004836-200307000-00012

[28] Ohkusa T, Kato K, Terao S, et al: Newly developed antibiotic combination therapy for ulcerative colitis: a double-blind placebo-controlled multicenter trial. Am J Gastroenterol, 2010; 105: 1820-1829. 20216533 10.1038/ajg.2010.84

[29] Ishikawa D, Sasaki T, Osada T, et al: Changes in intestinal microbiota following combination therapy with fecal microbial transplantation and antibiotics for ulcerative colitis. Inflamm Bowel Dis, 2017; 23: 116-125. 27893543 10.1097/MIB.0000000000000975

[30] Ishikawa D, Sasaki T, Takahashi M, et al: The microbial composition of Bacteroidetes species in ulcerative colitis is effectively improved by combination therapy with fecal microbiota transplantation and antibiotics. Inflamm Bowel Dis, 2018; 24: 2590-2598. 30124831 10.1093/ibd/izy266

[31] Kazemian N, Ramezankhani M, Sehgal A, et al: Author correction: The trans-kingdom battle between donor and recipient gut microbiome influences fecal microbiota transplantation outcome. Sci Rep, 2021; 11: 4546. 33608575 10.1038/s41598-021-82644-z PMC7895960

[32] Li SS, Zhu A, Benes V, et al: Durable coexistence of donor and recipient strains after fecal microbiota transplantation. Science, 2016; 352: 586-589. 27126044 10.1126/science.aad8852

[33] Okahara K, Ishikawa D, Nomura K, et al: Matching between donors and ulcerative colitis patients is important for long-term maintenance after fecal microbiota transplantation. J Clin Med, 2020; 9: 1650. 32486476 10.3390/jcm9061650 PMC7355579

[34] Knoll RL, Forslund K, Roat Kultima J, et al: Gut microbiota differs between children with inflammatory bowel disease and healthy siblings in taxonomic and functional composition: a metagenomic analysis. Am J Physiol Gastrointest Liver Physiol, 2017; 312: G327-G339. 28039159 10.1152/ajpgi.00293.2016

[35] Baruch EN, Youngster I, Ben-Betzalel G, et al: Fecal microbiota transplant promotes response in immunotherapy-refractory melanoma patients. Science, 2021; 371: 602-609. 33303685 10.1126/science.abb5920

[36] Davar D, Dzutsev AK, McCulloch JA, et al: Fecal microbiota transplant overcomes resistance to anti-PD-1 therapy in melanoma patients. Science, 2021; 371: 595-602. 33542131 10.1126/science.abf3363 PMC8097968

[37] Natarelli N, Aflatooni S, Boby A, Krenitsky A, Grichnik J: The gastrointestinal microbiome and immune checkpoint inhibitors: a review of human interventional studies among melanoma patients. J Drugs Dermatol, 2024; 23: 78-84. 38306142 10.36849/JDD.7674

[38] Reynolds HM, Bettini ML: Early-life microbiota-immune homeostasis. Front Immunol, 2023; 14: 1266876. 37936686 10.3389/fimmu.2023.1266876 PMC10627000

[39] Penders J, Stobberingh EE, van den Brandt PA, Thijs C: The role of the intestinal microbiota in the development of atopic disorders. Allergy, 2007; 62: 1223-1236. 17711557 10.1111/j.1398-9995.2007.01462.x

[40] Dominguez-Bello MG, Costello EK, Contreras M, et al: Delivery mode shapes the acquisition and structure of the initial microbiota across multiple body habitats in newborns. Proc Natl Acad Sci U S A, 2010; 107: 11971-11975. 20566857 10.1073/pnas.1002601107 PMC2900693

[41] Backhed F, Roswall J, Peng Y, et al: Dynamics and stabilization of the human gut microbiome during the first year of Life. Cell Host Microbe, 2015; 17: 690-703. 25974306 10.1016/j.chom.2015.04.004

[42] Houghton LA, et al: The role of diet and the microbiome in inflammation and autoimmunity. J Nutr Biochem, 2019; 70: 73-86.

[43] Korpela K, Helve O, Kolho K-L, et al: Maternal fecal microbiota transplantation in cesarean-born infants rapidly restores normal gut microbial development: a proof-of-concept study. Cell, 2020; 183: 324-334. e5. 33007265 10.1016/j.cell.2020.08.047

[44] Zeidan J, Fombonne E, Scorah J, et al: Global prevalence of autism: a systematic review update. Autism Res, 2022; 15: 778-790. 35238171 10.1002/aur.2696 PMC9310578

[45] Svoboda E: Could the gut microbiome be linked to autism? Nature, 2020; 577: S14-S15. 31996829 10.1038/d41586-020-00198-y

[46] Sanlier N, Kocabas S: The effect of probiotic, prebiotic and gut microbiota on ASD: a review and future perspectives. Crit Rev Food Sci Nutr, 2023; 63: 2319-2330. 34486891 10.1080/10408398.2021.1973957

[47] Leader G, Abberton C, Cunningham S, et al: Gastrointestinal symptoms in autism spectrum disorder: a systematic review. Nutrients, 2022; 14: 1471. 35406084 10.3390/nu14071471 PMC9003052

[48] Saurman V, Margolis KG, Luna RA: Autism spectrum disorder as a brain-gut-microbiome axis disorder. Dig Dis Sci, 2020; 65: 818-828. 32056091 10.1007/s10620-020-06133-5 PMC7580230

[49] Kang DW, Adams JB, Coleman DM, et al: Long-term benefit of microbiota transfer therapy on autism symptoms and gut microbiota. Sci Rep, 2019; 9: 5821. 30967657 10.1038/s41598-019-42183-0 PMC6456593

[50] Chen CC, Chiu CH: Current and future applications of fecal microbiota transplantation for children. Biomed J, 2022; 45: 11-18. 34781002 10.1016/j.bj.2021.11.004 PMC9133305

[51] Liptak R, Gromova B, Gardlik R: Fecal microbiota transplantation as a tool for therapeutic modulation of non-gastrointestinal disorders. Front Med (Lausanne), 2021; 8: 665520. 34557498 10.3389/fmed.2021.665520 PMC8452915

[52] Borsom EM, Lee K, Cope EK: Do the bugs in your gut eat your memories? Relationship between gut microbiota and Alzheimer’s disease. Brain Sci, 2020; 10: 814. 33153085 10.3390/brainsci10110814 PMC7693835

[53] Chok KC, Ng KY, Koh RY, et al: Role of the gut microbiome in Alzheimer’s disease. Rev Neurosci, 2021; 32: 767-789. 33725748 10.1515/revneuro-2020-0122

[54] Hazan S: Rapid improvement in Alzheimer’s disease symptoms following fecal microbiota transplantation: A case report. J Int Med Res, 2020; 48. 10.1177/0300060520925930 PMC7328362 32600151

[55] Park SH, Lee JH, Shin J, et al: Cognitive function improvement after fecal microbiota transplantation in Alzheimer’s dementia patient: A case report. Curr Med Res Opin, 2021; 37: 1739-1744. 34289768 10.1080/03007995.2021.1957807

[56] Xiaolan Z, Rumeng C, Yichen C, et al: Fecal Microbiota Transplantation: A Prospective Treatment for Type 2 Diabetes Mellitus. Diabetes Metab Syndr Obes, 2024; 17: 647-659. 38347911 10.2147/DMSO.S447784 PMC10860394

[57] Wang H, Lu Y, Yan Y, et al: Promising treatment for type 2 diabetes: fecal microbiota transplantation reverses insulin resistance and impaired islets. Front Cell Infect Microbiol, 2019; 9: 455. 32010641 10.3389/fcimb.2019.00455 PMC6979041

[58] Ng SC, Xu Z, Mak JWY, et al: Microbiota engraftment after faecal microbiota transplantation in obese subjects with type 2 diabetes: a 24-week, double-blind, randomised controlled trial. Gut, 2022; 71: 716-723. 33785557 10.1136/gutjnl-2020-323617

[59] Ding D, Yong H, You N, et al: Prospective study reveals host microbial determinants of clinical response to fecal microbiota transplant therapy in type 2 diabetes patients. Front Cell Infect Microbiol, 2022; 12: 820367. 10.3389/fcimb.2022.820367 PMC8990819 35402293

[60] Qiu XX, Cheng SL, Liu YH, et al: Fecal microbiota transplantation for treatment of non-alcoholic fatty liver disease: Mechanism, clinical evidence, and prospect. World J Gastroenterol, 2024; 30: 833-842. 38516241 10.3748/wjg.v30.i8.833 PMC10950639

[61] Yang Y, Schnabl B: Gut Bacteria in Alcohol-Associated Liver Disease. Clin Liver Dis, 2024; 28: 663-679. 39362714 10.1016/j.cld.2024.06.008 PMC11450261

